# Partial Analysis of the Capsid Protein (VP1) of Human Sapovirus Isolated from Children with Diarrhoea in Rural Communities of South Africa

**DOI:** 10.1155/2022/9928378

**Published:** 2022-06-02

**Authors:** Mpho Magwalivha, Jean-Pierre Kabue Ngandu, Afsatou Ndama Traore, Natasha Potgieter

**Affiliations:** Department of Biochemistry and Microbiology, Faculty of Science Engineering and Agriculture, University of Venda, Thohoyandou 0950, South Africa

## Abstract

**Background:**

Viral diarrhoea is a concern in acute gastroenteritis cases among children younger than 5 years of age. Sapovirus has been noted as an emerging causative agent of acute gastroenteritis worldwide. *Objective/Study Design*. The aim of this study was to characterize human sapoviruses targeting the VP1 (NVR and N-terminal) region. Twenty-five samples were randomly selected from 40 sapovirus-positive samples previously detected and analyzed for the VP1 region using the One-Step RT-PCR assay. The PCR products were subjected to Sanger sequencing analysis.

**Results:**

The polyprotein segment (NVR and N-terminal) was successfully amplified from 10/25 samples. Sapovirus GI.1 was the most predominant strain (6/10; 60%), followed by SV-GII.1 (2/10; 20%) and 10% of each GI.3 and GII.3.

**Conclusion:**

Through the partial analysis of the VP1 region, this study provides more data to add on the human sapovirus genetic characterization of circulating strains in South Africa, with the proposition of further analysis of sapovirus VP1 fragments for the viral structure and function.

## 1. Introduction

Human sapovirus (SV) as one of the leading causative agents of diarrhoea in young children is becoming notable worldwide [1, 2]. Information on the distribution of SV genotypes can give insights into the patterns of probable transmission, immunity amongst exposed people, relevant diagnosis of the circulating strains, and development of vaccine to regulate or eradicate virulent strains [3]. Human SV has four well-known genogroups, namely, genogroups I, II, IV, and V. Although SV-GI is the most detected genogroup followed by SV-GII, these genogroups are commonly associated with acute gastroenteritis across age groups and often detected in infants [2, 4–8]. There are few studies on the detection of human SV in South Africa which have been reported in different settings, that is, on rural outpatients [9], in a longitudinal study [10], on urban hospitalized children [11, 12], and on all age groups [13].

Human SV, a single-stranded positive-sense RNA virus classified in the *Caliciviridae* family, has a genome estimated to be 7.7* *kb in size. Amongst the three open reading frames (ORF-1, 2, and 3) documented, ORF-1 amongst other proteins contains the major capsid protein (VP1). The VP1 region is used for the classification of sapoviruses [14], and it is the most common targeted area for detection of this virus. Moreover, VP1 contains a segment which correlates with the genetic diversity and antigenicity of SV [4, 15, 16].

A variety of primers have been designed from previously accessible nucleotide sequences and tested for the detection of SVs [1, 15]. The capsid protein is described to have four regions, namely, *N*-terminal variable region (NVR) with 1–43 amino acid aa sequence containing 6.1% conserved residues, followed by the well-conserved section of the *N*-terminal region (*N*) with 44–285 aa sequence containing 40.5% conserved residues, a central variable region (CVR) with 286–441 aa sequence containing 5.9% conserved residues, and a last quarter of the *C*-terminal region (*C*) with 442–561 aa sequence containing 27.3% conserved residues [16].

The conserved amino acid motifs of SV are predicted to be positioned on VP1. Therefore, analysis of this protein may play a role in proper understanding of the diversity of strains that are circulating, for epidemiological surveillance to monitor emerging strains and controlling of pathogens posing serious illness among people. The detections of human SV have been reported worldwide, but mostly targeting a short sequence at the conserved RdRp/VP1 junction region of these viruses [2, 15]. This study aimed at reporting on the identification of human SV targeting a larger conserved region of the capsid protein (VP1).

## 2. Methods

### 2.1. Ethical Clearance and Consent

Ethical clearance for this study was obtained from the UNIVEN Research Ethics Committee (SMNS/18/MBY/02), and permission to collect samples was obtained from the Limpopo Provincial Department of Health and the District (Ref: 4/2/2). Written informed consent was obtained from all parents or guardians of participating children before stool sample collection.

### 2.2. Sample Selection

Twenty-five (62.5%) randomly selected and extracted RNA samples, from 40 samples which were previously identified as SV-positive [9], were subjected to further RT-PCR amplification targeting the fragments which form the capsid protein (VP1).

### 2.3. Amplification of Partial VP1 by RT-PCR

The One-Step RT-PCR (QIAGEN, Germany) kit was used for amplification of the selected SV-positive samples. Selected primers (Table 1) were used on the attempt to target region of the VP1 sequence [17]. The reagent mixture for SV amplification were as follows: 25 *µ*l of reaction volume containing 5 *µ*l of 5X One-Step RT-PCR buffer, 1 *µ*l of One-Step RT-PCR enzyme mix, 1 *µ*l of dNTP mix (containing 10 mM of each dNTP), 1.5 *µ*l of 0.6 *µ*M of each primer, 10 *µ*l of RNase-free water, and 5 *µ*l of the RNA sample. Amplification was done using the following conditions set on a T100 Thermal cycler (Bio-Rad, USA): reverse transcription at 50°C for 30 min; followed by initial PCR activation at 95°C for 15 min; then 39 cycles of three-step cycling (denaturation for 30 sec at 94°C, annealing for 30 sec at 53.5°C, and extension for 60 sec at 72°C); and final extension at 72°C for 10 min.

**Figure 1:**
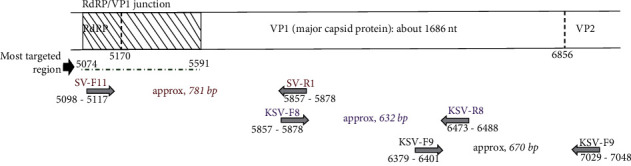
Schematic diagram of human sapovirus partial genomic organization and RT-PCR target regions. The diagram presents the most targeted RdRp/VP1 junction by RT-PCR pointed by a black arrow, and the location of primers targeting the complete VP1 segment shown by grey arrows on the binding location.

The location of part of the genome targeted for PCR amplification is important to understand the diversity and virulence of pathogens which are detected.  Figure 1 displays the targeted locations by selected primers aligned against the VP1 segment, for amplification of human sapoviruses. In previous studies, the most reported target region of the SV genome has been the RdRp/VP1 junction [15].

### 2.4. Sequencing

PCR products of the amplified fragments were directly purified with a master mix of ExoSAP (Nucleics, Australia). Using the same primers (Table 1), Sanger sequencing was performed on the ABI 3500XL Genetic Analyzer POP7TM (Thermo-Scientific). The obtained nucleotide of the successful sequenced amplicons were compared against those of the reference strains available in the NCBI GenBank, using BLAST available at https://www.ncbi.nlm.nih.gov/blast [18]. The reference strains with sequences of ≥709 nucleotides (SV-GI) and ≥644 (SV-GII) nucleotides were randomly selected among BLAST hits with >85% similarities on the query sequences of SV strains detected from this study. For confirmation of SV genotypes, the human calicivirus typing tool available at https://norovirus.ng.philab.cdc.gov [19] was used. Phylogenetic analysis was performed to check for close relatedness of human SV strains using MEGA 11 [20]. The confirmed nucleotide sequences were submitted to GenBank under the accession numbers OK180480–OK180489.

## 3. Results

### 3.1. RT-PCR Amplification

This study reports on 40% (10/25) successful amplification of partial VP1 using SV-F11 and SV-R1 primers, while other 15 samples failed to amplify. In addition, other pairs of primers used failed to amplify in all selected samples. The unsuccessful amplifications are suggested to be as a result of degraded or limited RNA genome copies. Sixty percent (6/10) of the results was identified as SV-GI.1, followed by 20% (2/10) SV-GII.1 and 10% of each GI.3 and GII.3 (Table 2). Of these 10 amplified samples, 60% (6) were obtained from outpatient children with diarrhoea and 40% (4/10) from hospitals based in the rural communities of Vhembe district, South Africa.

### 3.2. Sequence and Phylogenetic Analysis

**Figure 2:**
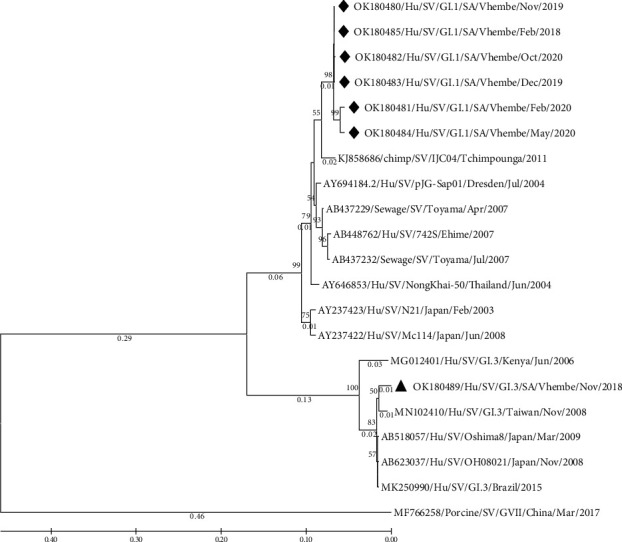
Phylogenetic analysis of the partial polyprotein of human SV-GI detected in Vhembe district (South Africa) and reference strains selected from the GenBank database. The percentage of replicate trees in which the associated taxa clustered together in the bootstrap test (1000 replicates) are shown next to the branches. A bar scale representing a genetic distance scale. The phylogenetic tree was deduced by the maximum likelihood method and the Kimura 2-parameter model using MEGA 11 [20, 21], based on a 709-nucleotide sequence fragment of the polyprotein (a VP1 segment) showing relationships of SV strains. The porcine SV (MF766258) was selected as an outgroup strain.

**Figure 3:**
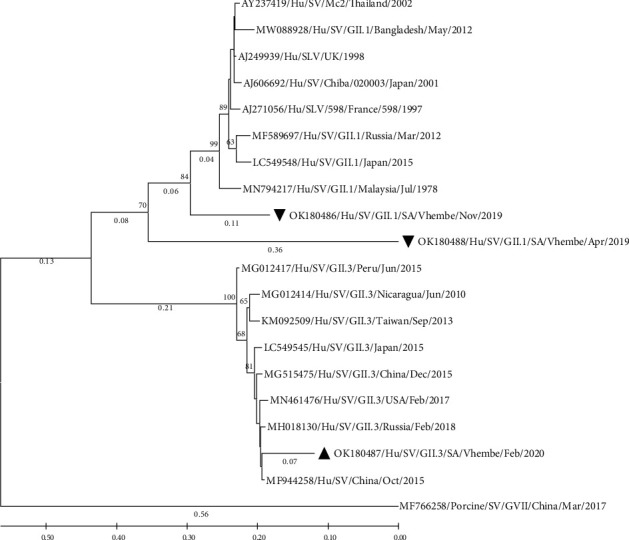
Phylogenetic analysis of the partial polyprotein of human SV-GII detected in Vhembe district, South Africa, and reference strains selected from the GenBank database. The percentage of replicate trees in which the associated taxa clustered together in the bootstrap test (1000 replicates) are shown next to the branches. A bar scale representing a genetic distance scale. The phylogenetic tree was deduced by the maximum likelihood method and the Kimura 2-parameter model using MEGA 11 [20, 21], based on a 644-nucleotide sequence fragment. The porcine SV (MF766258) was selected as an outgroup strain.

The sequences generated from this study were identified as the polyprotein segment. The human calicivirus typing tool gave a BLAST score of 75–99% capsid protein identity. A BLAST search gave a hint of 87–98% similarities of all featured sequences on a phylogenetic tree (Figures 2 and 3).

The phylogenetic tree (Figure 2) presents two distinctive clusters of genotypes, SV-GI.1 and SV-GI.3, showing relatedness of their strains. The detected SV-GI.1 genotypes (OK180480–OK180485) from this study presented an isolated cluster showing their close relatedness and a slight relatedness to a strain detected in Congo from a chimpanzee (KJ858686), with a common ancestor. The GI.1 strains detected in this study showed similarities of between 93 and 96.43% with KJ858686 strain on BLAST. The detected SV-GI.3 (OK180489) from this study clustered with a strain (MN102410) detected in Taiwan, and this strain had 97.38% identity on BLAST. Although other reference strains had similarities of between 90 and 98% (BLAST) with the detected strains from this study, they showed distinct clusters when rooted by a porcine SV strain (MF766258).

The phylogenetic tree (Figure 3) displays two distinctive clusters of genotypes, SV-GII.1 and SV-GII.3, showing their strains' relatedness. The detected GII.1 strains (OK180486 and OK180488) from this study did not cluster with any of the reference strains although sharing a common ancestor and similarity hints of >85% on BLAST. Within a GII.3 cluster, relatedness of a strain (OK180487) detected in this study with a strain (MF944258) detected in China is shown by a distinct cluster and these strains had a similarity of 89.62% on BLAST. Reference strains used on the phylogenetic tree (Figure 3) gave similarity hints of between 85% and 92.55% (BLAST) to the detected strains from this study, although they present distinct clusters from strains reported in this study.

## 4. Discussion

The detection of circulating human SVs has been previously reported based on the analysis of small fragments (especially the RdRp/VP1 junction) on the ORF1 segment of the viral genome [15]. In this study, the successful amplification of partial VP1 provides valuable data on circulating SV strains in South Africa. This study reports on 40% (10/25) partial sequencing of the VP1 (polyprotein) fragment of the human SV strain circulating in the rural areas of South Africa. It has been previously reported that single-stranded RNA is generally known to be very unstable, which may lead to difficulties of generating positive results [21]. This may explain the reason that some amplicons failed to generate successful sequence in this study.

The SV GI.1, reportedly associated with gastroenteritis [23], was noted as a dominate genotype (60%: 6/10) from this study. SV-GI.1 strains were detected from 66.7% (4/6) cases of patients admitted in hospitals and 33.3% (2/6) cases of patients in clinics (Table 2). However, other strains (SV-GII.1 (20%: 2/10), SV-GI.3 (10%: 1/10), and SV-GII.3 (10%: 1/10)) were detected from patients aided in clinics. SV-GI.1 seems to be the most widespread genotype, since it was also reported as the predominant strain in a study from Brazil [4], with other genotypes detected at a low rate similar to the findings of this study.

The ORF1 of SV is proposed to encode a polyprotein that is processed by the viral protease, resulting in manifestation of proteins needed for the viral genome's replication [14, 24]. In this study, the analysis of long amino acid sequences of NVR and N terminals was performed. It has been proposed that analysis of viral polyproteins provides understanding in mechanisms and clues leading to the drug design against viral diseases [24]. Moreover, the NVR is reportedly common to all SV genotypes [15, 16], and *N*-terminal has been identified as an area which can undergo significant conformational variation [14]. The analysis of the capsid protein region (VP1) is very crucial, since genotyping of SV is based on the capsid, which strongly correlates with the viral antigenic properties [16].

Sapovirus GI is known to be associated with severe diarrhoeal disease. Furthermore, GI.1 strains have previously been detected in acute gastroenteritis cases in studies conducted in Africa [23, 25, 26]. The phylogenetic analysis on this study showed a variability of strains detected. A discrete cluster of the detected strains (OK180480–OK180485) within genotype GI.1 suggests slight mutations on strains circulating in the Vhembe rural communities, South Africa (Figure 2). In addition, a slight relation of the cluster of GI.1 strains from this study with a strain (KJ858686) detected from a nonhuman host proposed a possibility of zoonotic transmission. Although close relatedness of human genogroups to the nonhuman genogroups have been predictable [27], more analysis on SV strains detected from human and other mammalians should be performed to confirm zoonotic transmission.

Sapovirus-GII is mostly associated with nonsevere diarrhoea, and it has been reportedly detected in diarrhoea cases in Africa [23, 25, 28]. Among other strains of genogroup-II, the most commonly detected is GII.1 as also reported in South Africa by Murray et al. [25]. The identified SV-GII.1 in this study clustered away from each other and reference strains, which could suggest possible mutations based on the occurred number of substitutions per site measured by the branch lengths (Figure 3).

A more successful detection rate of SVs has been commonly achieved by targeting the RdRp/VP1 junction [29, 30]. However, the analyzed segment in this study is reliable for accurate strain identification, since it is most variable and contains the maximum conserved residues on the VP1 sequence [15, 29]. From BLAST search list, there were no available data on SV strains previously reported in South Africa that could be used for phylogenetic relatedness. Moreover, analysis of a larger fragment on SV should be considered, as it creates a better possibility of genetic characterization by sequence analysis and comparison of strains around the globe. To our knowledge, this is the first study in South Africa to report on analysis of the large fragment (≥644 nucleotide long) of human sapovirus VP1.

## 5. Conclusion

Our study reports on partial analysis of VP1 protein (polyprotein), suggested to be responsible for viral protein folding, dimer formation, and viral particle assembly. Predominance of SV-GI.1 was determined in this study. Studies on the antigenicity and VP1 variations of SV are needed for accessing the role of SV as an emerging virulent agent associated with diarrhoeal diseases in young children. Analysis of SV's capsid protein should be carried out for epidemiological reference, to identify the diversity of virulent proteins produced and possibly to give insight into vaccine proposition.

## Figures and Tables

**Table 1:** Primers for amplification of VP1 segments [17].

PCR type	Primer	Sequence	Location	Product size (bp)
One-Step RT-PCR	SV-F11	GCY TGG TTY ATA GGT GGT AC	5098–5117	781
	SV-R1	CWG GTG AMA CMC CAT TKT CCA T	5857–5878	
	KSV-F8	ATG GAM AAT GGK GTK TCA CCW G	5857–5878	632
	KSV-R8	AGC CAG TGT GGC TGT GA	6473–6488	
	KSV-F9	GAC TTT GAC ACY AGT GGY TTT GC	6379–6401	670
	KSV-R9	CCA TTR ATG GAG AGG TCY CG	7029–7048	

**Table 2:** Positive samples by partial VP1 analysis.

Sample ID (admitted)	Collection date	Place coordinates	Sapovirus genotype	Accession numbers
R17 (Mph.clinic)	2019/11/29	22°38′58.6″S	GI.1	OK180480
		30°49′48.0″E		

R77 (Tsh.hospital)	2020/02/26	22°59′42.0″S	GI.1	OK180481
		30°24′52.7″E		

R95 (Sil.hospital)	2020/10/03	22°54′03.5″S	GI.1	OK180482
		30°11′37.3″E		

R26 (Tsh.hospital)	2019/12/18	22°59′42.0″S	GI.1	OK180483
		30°24′52.7″E		

R102 (Eli.hospital)	2020/05/03	23°09′16.6″S	GI.1	OK180484
		30°03′20.2″E		

Z01 (Xig.clinic)	2018/02/11	22°55′29.0″S	GI.1	OK180485
		30°43′21.7Ë		

R21 (Maj.clinic)	2019/11/20	23°13′17.6″S	GII.1	OK180486
		30°20′08.8″E		

R80 (Sil.clinic)	2020/02/28	22°54′03.2″S	GII.3	OK180487
		30°11′37.2″E		

R24 (Maj.clinic)	2019/04/12	23°13′17.6″S	GII.1	OK180488
		30°20′08.8″E		

Z31 (Mal.clinic)	2018/11/03	23°00′47.9″S	GI.3	OK180489
		30°42′05.2″E		

## Data Availability

All data supporting this results are included within the manuscript.
